# Tolerance to Cadmium of *Agave lechuguilla* (Agavaceae) Seeds and Seedlings from Sites Contaminated with Heavy Metals

**DOI:** 10.1155/2013/167834

**Published:** 2013-12-23

**Authors:** Alejandra Méndez-Hurtado, René Rangel-Méndez, Laura Yáñez-Espinosa, Joel Flores

**Affiliations:** ^1^Instituto de Ingeniería y Tecnología, Universidad Autónoma de Ciudad Juárez, 32310 Ciudad Juárez, CHIH, Mexico; ^2^División de Ciencias Ambientales, Instituto Potosino de Investigación Científica y Tecnológica, 78210 San Luis Potosí, SLP, Mexico; ^3^Instituto de Investigación de Zonas Desérticas, Facultad de Ingeniería, Universidad Autónoma de San Luis Potosí, 78377 San Luis Potosí, SLP, Mexico

## Abstract

We investigated if seeds of *Agave lechuguilla* from contaminated sites with heavy metals were more tolerant to Cd ions than seeds from noncontaminated sites. Seeds from a highly contaminated site (Villa de la Paz) and from a noncontaminated site (Villa de Zaragoza) were evaluated. We tested the effect of Cd concentrations on several ecophysiological, morphological, genetical, and anatomical responses. Seed viability, seed germination, seedling biomass, and radicle length were higher for the non-polluted site than for the contaminated one. The leaves of seedlings from the contaminated place had more cadmium and showed peaks attributed to chemical functional groups such as amines, amides, carboxyl, and alkenes that tended to disappear due to increasing the concentration of cadmium than those from Villa de Zaragoza. Malformed cells in the parenchyma surrounding the vascular bundles were found in seedlings grown with Cd from both sites. The leaves from the contaminated place showed a higher metallothioneins expression in seedlings from the control group than that of seedlings at different Cd concentrations. Most of our results fitted into the hypothesis that plants from metal-contaminated places do not tolerate more pollution, because of the accumulative effect that cadmium might have on them.

## 1. Introduction

Availability of heavy metals in soil depends on pH, clay content, and organic matter content, as well as on cation and/or anion exchange capacity [1]. Toxicity for plants depends on heavy metals availability; there are “nonaccumulator species” that have less tolerance to heavy metals [2]; “tolerant species” that grow on soils with high concentrations of heavy metals without any apparent severe damage [3]; there are also “accumulator species” that have the ability to absorb and accumulate certain concentrations of heavy metals in their tissues [4, 5], and there are some species known as “hyperaccumulators” that take up particularly high amounts of a toxic substance, usually a metal or metalloid, in their shoots during normal growth and reproduction [6].

Some plant species have developed detoxification mechanisms against stress caused by heavy metals, such as Cd, producing small proteins rich in cysteine that chelates metals [7, 8]. Among the heavy metal-binding ligands in plant cells the phytochelatins (PCs) and metallothioneins (*MTs*) are the best understood [6–10]. Some plants, such as the hyperaccumulators do not detoxify metals by huge accumulation of PCs but by sequestering them into the vacuoles [11, 12].

Cadmium is a nonessential element that can be highly toxic for nonhyperaccumulator plants at low concentrations and has been ranked number 7 among the top 20 toxins [10]. Cd can cause strong oxidative stress, thereby inactivating PSII and photosynthetic electron transport [13]. This element diminishes radicle growth of seedlings and the length of roots of older plants; it also diminishes biomass production, stomatal conductance, transpiration rate, and other metabolic activities [9–11, 13, 14]. Cd also interferes with the capture, transport, and use of several micronutrients such as Fe, Mn, and Zn [10].

In the municipality of Villa de la Paz in the state of San Luis Potosí (México), where a Pb-Zn-Ag (Cu-Au) skarn ore system has been mined for more than 200 yr, a high grade contamination for As, Cu, Zn, Pb, and Cd related to fluvial dispersion of mine waste through streams has been found [15]. The ecological and health risk in this remains to be studied.

*Agave lechuguilla* occurs in semiarid regions from the Chihuahuan Desert, especially at the Central Mexico where the pollution generated by the large-scale mining industry generates mine tailings that are highly polluted with heavy metals and metalloids [16]. If *A. lechuguilla* is tolerant to heavy metal toxicity remains unknown, but it contains two steroidal saponins (yuccagenin and ruizgenin) which can be used as natural chelating agents to remove heavy metals such as Cr, Cd, Cu, Pb, and Zn from soil and waste [17].

In a metal-polluted site the *A. lechuguilla* response could vary; thus, we expected one of the following three possibilities: (i) the individuals having resistance to cadmium have descendants or viable seeds so that a selection process for resistance has taken place in contaminated sites and plants from such sites grow well under Cd presence; (ii) plants from metal-contaminated sites have overcome an initial shock to Cd and are hence more likely to grow under the presence of more Cd; or (iii) plants from metal-contaminated places do not tolerate more pollution, because of the accumulative effect that cadmium might have on them. The aim of this study was to determine the effect that Cd has on seeds and seedlings of *A. lechuguilla* from sites with different contamination levels by heavy metals, in order to determine if seeds from contaminated sites by heavy metals are more tolerant to contamination than seeds from noncontaminated sites.

## 2. Materials and Methods

### 2.1. Studied Species and Seed Collection

*Agave lechuguilla* Torr. (Agavaceae) seeds were collected from two places in the state of San Luis Potosi, Mexico. One site is located in the municipality of Villa de la Paz, which is a contaminated area with heavy metals from mining activities [15] and the second, is in Villa de Zaragoza a site and considered as uncontaminated without mining activity. The seeds were collected by hand harvesting the fruits and removing the seeds, from at least 10 mother plants, in Spring of 2010).

### 2.2. Heavy Metals in the Soil

Heavy metals in soils of Villa de Zaragoza were determined and compared with those from the literature of Villa de la Paz [15]. A sample of 5 g of soil was placed in 15 mL Eppendorf tubes and then 10 mL of acidic water were added. The solution was adjusted to a pH of five with nitric acid in order to determine the metals and their concentration that could be available during seed germination and seedling growth during rainfall. The Eppendorf tubes were centrifuged at 6000 g units for 10 minutes. The supernatant was analyzed by inductively coupled plasma (ICP mode) to determine the concentrations of metals in the soil. There were four replicates for each test and the average was used.

### 2.3. Chemical Functional Groups Determination by FTIR Spectroscopy in Seeds

Fourier transform infrared (FTIR) spectroscopy analyses were conducted on seeds that were first dried at 70°C for 17 h and then grinded before analysis. These analyses allowed identification of chemical functional groups, such as carboxyl, amines, amides, aldehydes, and sulphur, with which Cd could interact. These experiments were conducted by a Thermo Nicolet FTIR 6700 spectrometer by the attenuated total reflectance (ATR) method. The spectra were obtained from 650 to 4000 cm^−1^ with 6 cm^−1^ resolution, collected 16 times, and corrected for background noise [18].

### 2.4. Determination of Concentration of Metals in Seeds by Inductively Couple Plasma (ICP Mode)

Prior to analysis, seeds were dried at 45°C for 24 hours and grounded. A sample of 0.5 g of each seed was acid digested (for 5 hours at 70°C) by 0.1 mL of aqua regia (1 part of HNO_3_ plus 3 volumes of HCl) for each milligram of sample. Hydrogen peroxide (H_2_O_2_) was added at the end of digestion in order to oxidize the remaining organic matter. The remaining solution with a pH near zero was then analyzed by ICP to determine the concentration of metals [19].

### 2.5. Seed Viability

Tetrazolium tests were conducted to evaluate the viability of seeds, as suggested by C. Baskin and J. M. Baskin [20]. Tetrazolium at 2% in deionized water was prepared in darkness to avoid the degradation of tetrazolium, and then 100 seeds from each locality were placed in this solution in a beaker, previously covered with aluminum foil to prevent exposure to light. Seeds were rinsed and placed in distilled water prior to microscopic analysis; those seeds with a pigmented red embryo were determined to be viable.

### 2.6. Effect of Cd on Seed Germination and Seedling Growth

The treatments consisted of five experimental units with 20 seeds per Petri dish, to which 2 mL of different initial concentration of Cd, from Cd(NO_3_)_2_·4H_2_O, was added as the only substrate. There were five replicates for each treatment. The pH of the Cd solutions was adjusted to five in order to determine the Cd uptake by seeds and seedlings during their germination and growth, respectively. Seven initial Cd concentrations were studied (1, 10, 20, 40, 60, 80, and 100 mg/L). A control (0 mg/L) was conducted in this series of experiments, in which deionized water was used.

The seeds were placed to germinate under a 12 h light by 12 h dark photoperiod at 25°C in an incubator (plant ICP-19d-c/iv Lumistell). Variables evaluated were germination percentage, radicle length (in mm), shoot dry weight (biomass in mg), and quantum efficiency of photosystem II. To evaluate seedling growth variables we used five plants (one by Petri dish) considered as replicates for each treatment. A portable pulse amplitude modulation fluorometer (Mini-PAM; H. Walz, Effeltrich, Germany) was used to measure the quantum efficiency of photosystem II.

### 2.7. Effect of Cd on the Anatomical Structure of Seedlings

Seedlings (*n* = 5 by treatment) were harvested after two weeks of growth under different Cd treatments. They were fixed in glutaraldehyde 2%, dehydrated with an ethanolic series, and embedded in Glycol Methacrylate (Technovit 7100). Transverse sections (2 *μ*m thick) were obtained with a rotary microtome (Leica RM 2125RT) and stained with brilliant cresyl blue 1% [21]. The observations of these sections were made with a light microscope (Leica DM 2000).

### 2.8. Cd Sorption by Seedlings

After germination experiments, seedlings (*n* = 5 by treatment) were harvested and their metals concentration was determined as follows. Leaves were washed with deionized water to remove any surface element that may cause interference. This was followed by drying seedlings at 45°C for 24 hours. After drying, the leaves were ground in an agate mortar and then acid digested. Following acid digestion, the products were analyzed by ICP mode to obtain the concentrations of Cd in leaves.

### 2.9. Metallothioneins

RNA extraction was done following Qiagen (2001). With this methodology, small RNAs such as 5.8S RNA, 5S RNA, and tRNA, of approximately 160, 120, and 70–90 nucleotides in length, respectively, are not isolated, giving more scope to the removal of messenger RNAs.

In order to evaluate the metallothionein expression in *A. lechuguilla* seedlings under Cd stress, seven initial Cd concentrations (0, 1, 10, 40, 60, 80, and 100 mg/L) were studied (*n* = 5 by treatment). We used SuperScript II Kit First-Strand Synthesis System for RT-PCR (Invitrogen, Carlsbad, CA). In each PCR reaction of 50 mL 1 mL of cDNA was added, using specific primers to amplify the actin 1 gene as an internal control of loading. Prior to this, specific primers were designed for the metallothionein gene of *A. lechuguilla* by Primer Select program of Lasergene (DNASTAR). The level of gene expression in each sample of *A. lechuguilla* was calculated based on the intensity of the band through the analysis software Quantity One 1D 4.5 (BIO-RAD, Hercules, CA.), carrying out a normalization process through the expression of an actin gene 1. The program for the amplification conditions were an initial cycle of 95°C for 5 min, followed by 95°C for 30 s (denaturation), 60°C for 1 min (alignment), and 72°C for 1.5 min (extension), 28 cycles. Amplified products were separated by electrophoresis on agarose gels with 1% 1X TAE stained with EtBr and photodocumented.

Differential expression of the gene transcript metallothionein (*MT*) of *A. lechuguilla* was measured by selecting seedlings to assess the accumulation of this transcript by RT-PCR. To evaluate the gene expression two independent experiments were performed by triplicate. The conditions of PCR reactions were optimized to avoid saturation in the accumulation of PCR products, maintaining a linear relationship to the original levels of the transcript in all samples. The intensities of the signals were quantified and the values were normalized with the housekeeping gene encoding an actin using 30 cycles of PCR.

### 2.10. Statistical Analysis

To determine differences in germination percentage, radicle length, seedling dry weight (biomass), and quantum efficiency of photosystem II among Cd treatments and sites, two-way ANOVAs were used, with Cd treatments (eight levels) and site (two levels: Villa de la Paz and Villa de Zaragoza) as main factors.

The statistical analyses were conducted by using the statistical program SAS [22], with *α* = 5%. The data of all response variables were transformed with natural logarithm to normalize their distribution, except for the germination percentages for which arcsine square root transformation was used [23].

## 3. Results

### 3.1. Heavy Metals in Soils and Seeds

Soil from Villa de Zaragoza contained copper and zinc, but at lower concentrations than soil from Villa de la Paz soil (Table 1). Seeds from Villa de la Paz had high contents of trace elements such as Al, Cd, Cu, Si, Sr, and Zn. Seeds from Villa de Zaragoza had Al, Cu, and Zn, but at lower concentrations than those found in the seeds of Villa de la Paz (Table 1).

Following FTIR analyses of seeds from Villa de la Paz and Villa de Zaragoza, we found that seeds from both sites showed the characteristic bands of chemical functional groups such as alkanes (1000–800 cm^−1^), primary and secondary amines (3300–3000 cm^−1^), amides (1700–1670 cm^−1^), carboxyls (1740–1210 cm^−1^), aldehydes (1680–1675 cm^−1^), and some sulfur containing groups (695–900 cm^−1^) that could adsorb and/or chelate metal cations.

### 3.2. Seed Viability and Effect of Cd on Seed Germination and Seedling Growth of Agave lechuguilla

Villa de Zaragoza seeds had higher viability (80%) than those from Villa de la Paz (45%). Germination was similar at all cadmium concentrations inside each site (Table 2); however, there were differences between sites for germination. Seed germination was affected by the site factor (*F* = 482.30; *P* < 0.0001) in that seeds from Villa de Zaragoza germinated more (76.0 ± 1.69%) than seeds from Villa de la Paz (26.63 ± 1.44%). Germination was not affected by the treatment factor (*F* = 1.46; *P* > 0.05), nor by the site X treatment interaction (*F* = 0.31; *P* > 0.05).

Radicle length was affected by the treatment factor (*F* = 45.81; *P* < 0.0001) in that shorter radicles were found for higher concentrations of cadmium in the seeds (Table 3). Radicle length was also affected by site factor (*F* = 176.09; *P* < 0.0001) in that seedlings from Villa de Zaragoza had higher radicle length (1.46 ± 0.03 cm) than those from Villa de la Paz (1.08 ± 0.02 cm), and also by the site X treatment factor (*F* = 7.37; *P* < 0.0001) in that radicle length was higher for Villa de Zaragoza seedlings in most Cd treatments.

Seedling biomass was affected by site factor (*F* = 2.60; *P* < 0.01) in that seedlings from Villa de Zaragoza had heavier biomass (2.4 ± 0.01 mg) than seedlings from Villa de la Paz (2.1 ± 0.02 mg). Seedling biomass was not affected by the treatment factor (*F* = 0.75; *P* > 0.05), nor by the site X treatment interaction (*F* = 1.25; *P* > 0.05).

The quantum efficiency of photosystem II was not affected by treatments (*F* = 1.21; *P* > 0.05), sites (*F* = 3.11; *P* > 0.05), or the site X treatment interaction (*F* = 1.54; *P* > 0.05). All values were close to 0.8.

### 3.3. Cadmium in Leaves and Functional Groups

**Figure 1:**
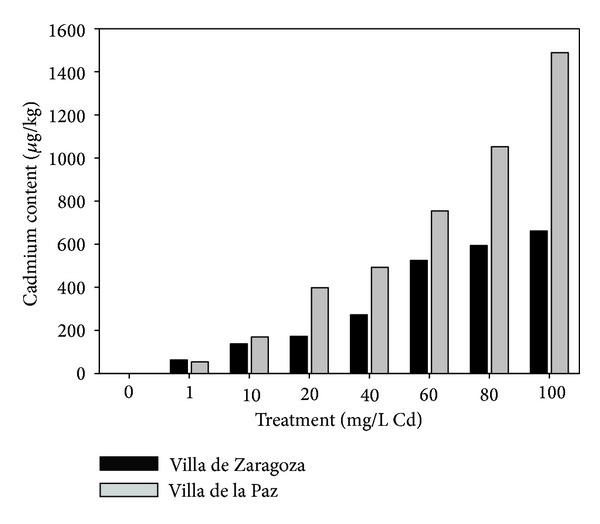
Cadmium concentration in *Agave lechuguilla* seedlings from two sites, Villa de la Paz (contaminated site) and Villa de Zaragoza (noncontaminated site).

**Figure 2:**
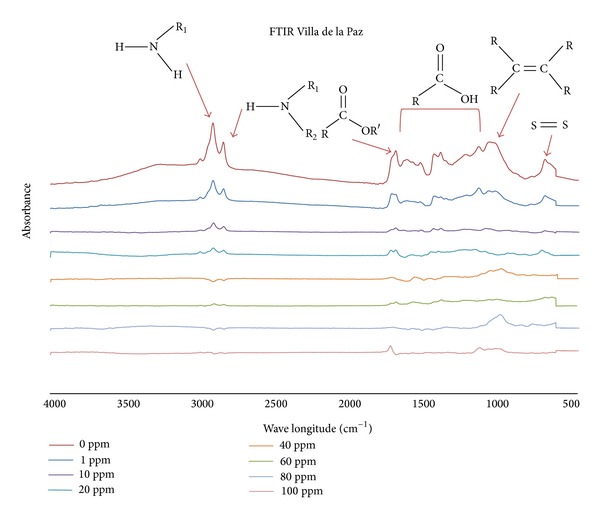
FTIR analyses of *A. lechuguilla* seedlings from Villa de la Paz treated with different Cd concentrations. Arrows indicate the peak for specific functional groups. The bracket indicates the wavenumber at which carboxyl groups can be found.

**Figure 3:**
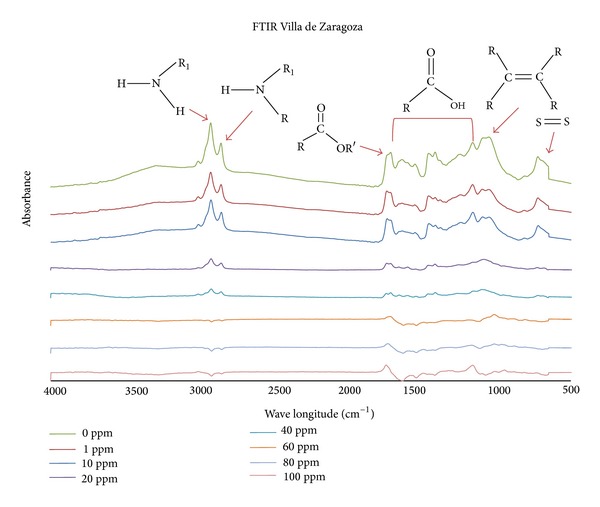
FTIR analyses of *A. lechuguilla* seedlings from Villa de Zaragoza treated with different Cd concentrations. Arrows indicate the peak for specific functional groups. The bracket indicates the wavenumber at which carboxyl groups can be found.

Higher cadmium concentration resulted in more cadmium accumulated in seedlings. The leaves of seedlings from Villa de la Paz had more cadmium than those from Villa de Zaragoza (Figure 1). In addition, FTIR results showed that peaks attributed to functional groups as amines, amides, carboxyl, and alkenes tended to disappear as increasing the concentration of cadmium (Figures 2 and 3). Samples from the contaminated site, Villa de la Paz, showed less concentration of carboxylic and aldehyde groups that could be attributed to the interaction of these groups with metals presented in Table 1.

### 3.4. Metallothioneins (MTs) in Leaves

**Figure 4:**
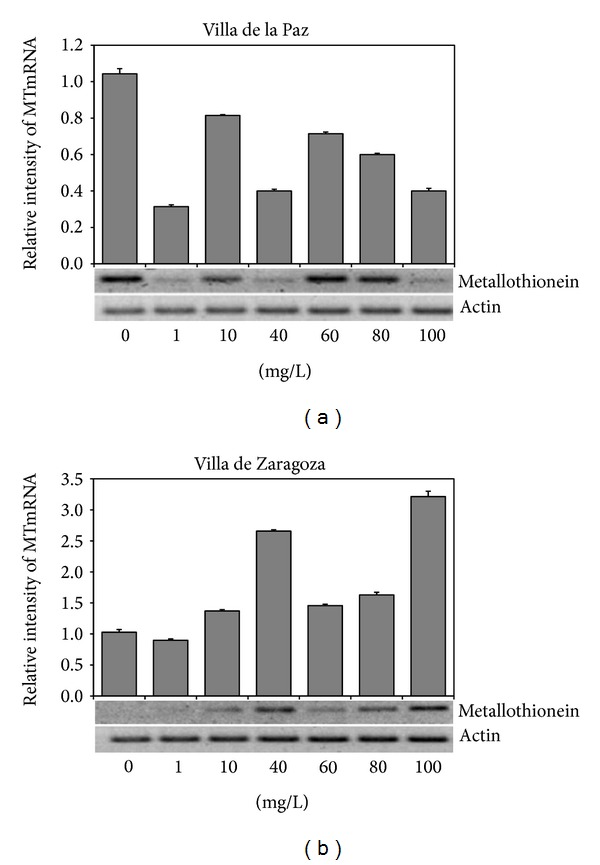
Differential expression of metallothionein (*MT*) gen in *Agave lechuguilla* seedlings from two sites, Villa de la Paz (contaminated site) and Villa de Zaragoza (noncontaminated site).

The leaves of seedlings from the contaminated place (Villa de la Paz) showed a higher *MTs* expression in the control group than those of seedlings treated with different Cd concentrations. In contrast, leaves form seedlings from the noncontaminated place showed slightly higher *MTs* with higher Cd concentrations (Figure 4).

### 3.5. Seedling Anatomy

Seedlings treated with cadmium showed various symptoms of toxicity: plumula tilting, cell senescence, chlorosis, delay in germination, and delay in radical growth, which were accentuated when increasing cadmium concentration, being 100 mg/L the most harmful concentration. These symptoms were more pronounced in the seedlings of Villa de la Paz.

**Figure 5:**
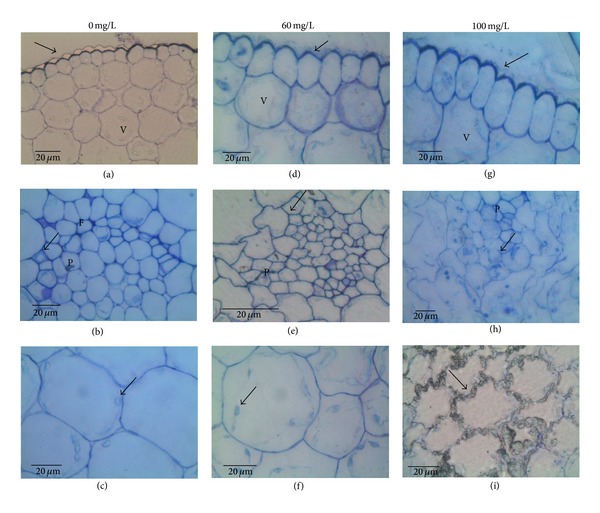
Transverse sections of *Agave lechuguilla* seedlings of Villa de Zaragoza site. (a), (b), (c) 0 ppm; (d), (e), (f) 60 ppm; (g), (h), (i) 100 ppm. Epidermal cells showing cell walls with normal thickening (arrow) and at (a) regular round form; (d) irregular elongated form; (g) regular elongated form. Vascular bundle with xylem vessels (arrow) (b) normal; (e) and (h) premature lignified. Cortex parenchyma cells with starch grains (arrow) (c) and (f) scarce; (i) abundant with a breakdown of the cells. v = vacuole; p = procambium. The analysis of this section was made with a light microscope (Leica DM 2000). Bars = 20 *μ*m.

**Figure 6:**
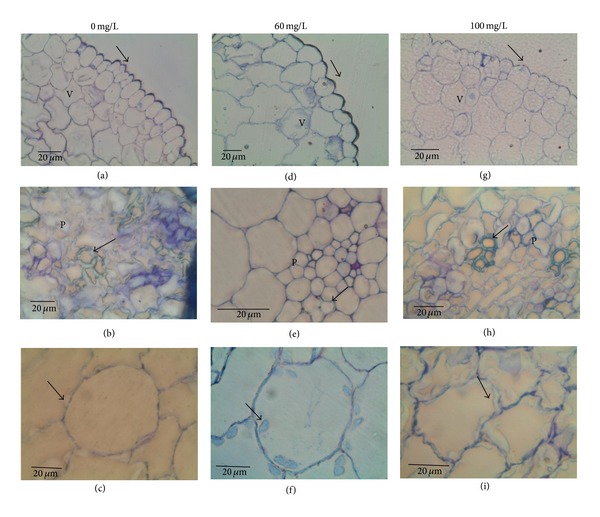
Transverse sections of *Agave lechuguilla* seedlings of Villa de la Paz site. (a), (b), (c) 0 ppm; (d), (e), (f) 60 ppm; (g), (h), (i) 100 ppm. Epidermal cells showing irregular form and periclinal cell walls (arrow) with (a) and (d) normal thickening; (g) thinner. Vascular bundle with xylem vessels (arrow) (b) normal; (e) and (h) premature lignified. Cortex parenchyma cells with starch grains (arrow) (c) and (f) scarce; (i) abundant with a breakdown of the cells. v = vacuole; p = procambium. The analysis of this section was made with a light microscope (Leica DM 2000). Bars = 20 *μ*m.

Figures 5 and 6 show the anatomical sections of seedlings from Villa de Zaragoza and Villa de la Paz, respectively. In general, the higher the concentration of cadmium the greater the damage in cells. Treated seedlings with different cadmium concentrations showed alterations in epidermal cells, xylem vessels, and cortex parenchyma cells. The epidermis was formed by a uniform row of regular rounded to elongated cells with thick anticlinal walls and thicker outer periclinal wall. This trend was maintained in the epidermis of the seedlings of Villa de Zaragoza, while for seedlings of Villa de la Paz the anticlinal and periclinal walls were thinner as the concentration of cadmium increased.

In both sites, the vascular bundles near the apical meristem broke down with increasing cadmium concentration. A premature lignification of xylem vessels was also observed at higher cadmium concentrations, losing their regular shape. The cortex parenchyma cells were thin walled and regular rounded, with abundant intercellular spaces, but as the concentration of cadmium increased parenchyma cells were less regular and became angular, reducing the intercellular spaces. At the highest cadmium concentration, the parenchyma cells were reduced in size and irregular in form and showed breakdown of the cells, and the intercellular spaces were absent. Longer starch granules in cells of seedlings from both Villa de Zaragoza and Villa de la Paz were found for higher concentrations of cadmium. However, starch granules in Villa de la Paz seedlings were larger than those from Villa de Zaragoza in the control treatment.

## 4. Discussion

In this study, contrasting results were obtained from a contaminated site by heavy metals, Villa de la Paz, and a nonpolluted site, Villa de Zaragoza. Soil chemical analysis showed that there was no high heavy metal contamination in Villa de Zaragoza, perhaps because this area does not have mining or industrial activity. However, the Villa de la Paz site, a mining site for over 200 years, showed high concentrations of cadmium, copper, arsenic, lead, and zinc [15]. Seeds from the contaminated site had high content of heavy metals such as Al (6.833 *μ*g/g), Cd (2.982 *μ*g/g), Cu (6.574 *μ*g/g), Si (7.866 *μ*g/g), Sr (1.356 *μ*g/g), and Zn (10.533 *μ*g/g). Two of them had a higher concentration than the elemental concentrations of the “standard reference plant”: Cd (0.05 *μ*g/g) and Cu (0.2 *μ*g/g) [24]. In contrast, Villa de Zaragoza seeds contained only aluminum, copper, and zinc, but in lower concentrations than those found in Villa de la Paz.

We had three hypotheses on the *A. lechuguilla* responses for the metal-polluted site, but most of our results fitted the hypothesis that plants from metal-contaminated places do not tolerate more pollution, because of the accumulative effect that cadmium might have on them. In general, both viability and germination were higher in seeds from the nonpolluted site than in the contaminated one. These findings are in agreement with Kranner and Colville [25], who suggested that seeds from plants exposed to high concentrations of heavy metals have low viability. However, our seed germination results are in contrast to findings by Haque et al. [26], who found that *Prosopis* sp. seeds from a contaminated site were adapted to pollution and had higher germination.

Other findings confirming the hypothesis that plants from metal-contaminated places do not tolerate more pollution are that biomass and radicle length were higher for seedlings from the noncontaminated site than for seedlings from the metal-contaminated site in most Cd treatments. Similar results were found by Buendía-González et al. [27], who found that smaller *Prosopis laevigata* seedlings with fewer leaves and secondary roots were produced by the effect of Cr(VI) and Cd(II).

The leaves of seedlings from Villa de la Paz had more cadmium than those from Villa de Zaragoza. FTIR results showed that samples from the contaminated site, Villa de la Paz, showed less concentration of carboxylic and aldehyde groups that could be attributed to the interaction of these groups with metals. They also showed that peaks attributed to functional groups as amines, amides, carboxyl, and alkenes tended to disappear with increasing cadmium concentration. These findings indicate that cadmium interacts with these functional groups, because cadmium has been found to bind to oxygen and nitrogen groups, such as amino and carboxyl groups [28].

The carboxyl functionality is the main group responsible for binding Cd for *Atriplex canescens*, another desert plant [18]. For *A. lechuguilla* seedlings, it is likely that carboxyl and nitrogen containing groups interact with cadmium through electrostatic and van der Waals forces. Cadmium could also form complexes with molecules in cells, for example, with metallothioneins which occur by stress. Thus, cadmium complexes with metallothionein or other molecules could be distributed throughout the seedling and remain in its structure, as reported by Clemens [29].

There are studies that confirm the metallothionein expression when seedlings are stressed by metals such as cadmium [25]. Our results showed a strong expression of *MT* in the control sample for seedlings from Villa de la Paz, the contaminated site. However, the expression of *MT* was lower than the control in seedlings exposed to various concentrations of cadmium. Such a response may be due to the high concentration of heavy metals in seeds of this site, so that seedlings accumulated more cadmium than those from Villa de Zaragoza. The stress by cadmium was possibly too much and the time given to seedlings to produce more *MTs* was short. Conversely, there was a gradual increase of *MTs* for seedlings of Villa de Zaragoza as the concentration of cadmium increased. This result is similar to that obtained by Cobbett and Goldsbrough [7] in *Arabidopsis* seeds under different concentrations of cadmium and zinc.

The anatomical structure of the cells also presented differences when treated by cadmium. Malformed cells were found (i.e., epidermal cells), likely because the epidermal cells are the first tissues that interact with the metal. We also found that at high concentration, the cells had a larger vacuole where they store cadmium, similar to that reported by Clemens [29]. Sites also showed differences, because the higher anatomical damage was found in seedlings from Villa de la Paz, where at 100 ppm of cadmium the epidermal cells were disorganized and had malformations. In contrast, for the Villa de Zaragoza seedlings at 100 ppm of cadmium, the epidermis was well organized and aligned, although it was malformed in some cells. High concentrations of cadmium have led to the loss of cell turgor, accompanied with intercellular air spaces, irregular-shaped epidermal and cortical cells, in willow (*Salix fragilis*) and poplar (*Populus* x *euramericana* (clone I-214)) [30].

Other response variables were also affected by cadmium treatments, but the site had no influence; that is, for seedlings from both sites, the higher the concentration of cadmium the greater the damage in cells. Seedlings with different cadmium concentrations showed alterations in epidermal cells, xylem vessels, and cortex parenchyma cells. The epidermis was formed by a uniform row of regular rounded to elongated cells with thick anticlinal walls and thicker outer periclinal wall. This trend was maintained in the epidermis of the seedlings of Villa de Zaragoza; for seedlings of Villa de la Paz the anticlinal and periclinal walls were thinner as the concentration of cadmium increased.

For seedlings from both sites, we found that the vascular bundles near the apical meristem broke down with increasing cadmium concentration. A premature lignification of xylem vessels was also observed at higher cadmium concentrations, losing their regular shape. The cortex parenchyma cells were thin walled and regular rounded, with abundant intercellular spaces, but as the concentration of cadmium increased parenchyma cells were less regular and became angular, reducing the intercellular spaces. At the highest cadmium concentration, the parenchyma cells were reduced in size and irregular in form, showing breakdown of the cells and the absence of intercellular spaces.

Cadmium can cause strong oxidative stress thereby inactivating PSII (quantum efficiency of photosystem II) and the photosynthetic electron transport [13]. However, Cd did not significantly affect the quantum efficiency of photosystem II of the *A. lechuguilla* seedlings at high concentrations. It is possible that cadmium interfered with other metabolic processes of the plants rather than with the thylakoid membranes of the photosynthetic apparatus [31].

The seedlings treated with high concentrations of cadmium showed abnormal growth (“bending”) on the part of the plumule. This result was because the xylem did not develop properly and there was a premature lignification of the xylem [31]. The xylem is a supporting structure for the seedlings and when this is not properly developed it causes abnormal growth of seedlings [32]. Other responses such as cellular senescence, chlorosis, and reduced seedling growth were found in *Agave lechuguilla* seedlings at the highest concentration of cadmium, which is similar to findings of several authors for seedlings of diverse species [33–37].

Cadmium was found to interfere in the vascularization of the seedlings at high concentrations of cadmium, because the xylem vessels had smaller diameter than seedlings in the control treatment. This is because when seedlings are under stress, the xylem vessels lignify prematurely, preventing normal development and full expansion [33, 38]. The xylem showed a greater damage than the phloem in presence of cadmium, because this metal enters directly into the xylem in the form of Cd^2+^ or as a complex with some proteins (metallothioneins, phytochelatin, or GSH), while Cd comes to the phloem together with sugar, with no effect on phloem [29]. Perhaps cadmium is entering via symplastic pathway in the seedlings until reaching the xylem. Then cadmium might incorporate with the xylem (joining photosynthates, sugars and other compounds) and finally reach the phloem [39, 40].

## 5. Conclusions

This study integrates the various disciplines in order to know if *Agave lechuguilla* seeds and seedlings from contaminated sites by heavy metals were more tolerant to Cd ions than those from noncontaminated sites. *Agave lechuguilla* seeds from Villa de la Paz (a contaminated site with heavy metal) had reduced viability, probably due to the higher concentration of metals present in their tissues compared with the seeds of Villa de Zaragoza. In addition, seeds from Villa de la Paz had lower germination, and their seedlings had reduced shoot biomass and radicle length, as well as greater accumulation of cadmium. However, the quantum efficiency of photosystem II was not affected by Cd treatments.

The metallothionein expression was greater in seedlings from Villa de la Paz. In the seedlings of Villa de Zaragoza, MT expression was not found in the control seedlings but only in those from treatments with cadmium. Contamination of soils from Villa de la Paz was shown to stress seedlings and cause the expression of *MTs*.

High concentrations of cadmium produced more interactions with functional groups belonging to different structures of molecules such as *MTs*, inhibiting the expression of these groups. Seedlings of Villa de la Paz (from a contaminated site) presented the greatest anatomical damage when increasing the cadmium concentration, for example, irregular-shaped epidermal cells and thinner epidermal cell wall in periclinal orientation. Cadmium pollution adversely affected seedling growth of *A. lechuguilla*. Taking into account the metal concentrations in the contaminated soils and the effects of the cadmium on the seedling development of *A. lechuguilla*, we conclude that cadmium might limit plant colonisation in contaminated sites with heavy metals.

## Figures and Tables

**Table 1:** Metals in soils, at pH 5, and in *Agave lechuguilla* seeds from a contaminated site (Villa de la Paz, SLP) and from a noncontaminated site (Villa de Zaragoza, S.L.P.).

Elements	Villa de Zaragoza (mg/kg) in soil	Villa de la Paz (mg/kg)* in soil	Villa de Zaragoza (mg/kg) in seeds	Villa de la Paz (mg/kg) in seeds	Detection limit of each element (mg/L)
Al	ND	ND	0.1696	6.833	0.045
As	ND	939	ND	ND	0.02
Cd	ND	16	ND	2.982	0.0034
Cu	0.0149	440	3.36	6.574	0.0054
Pb	ND	518	ND	ND	0.042
Si	ND	ND	ND	7.866	0.026
Sr	ND	ND	ND	1.356	0.0077
Zn	20.293	763	5.633	10.533	0.0018

ND: nondetected. Asterisks * indicate values reported by Razo et al. (2004) [15].

**Table 2:** Seed germination ± standard error of *Agave lechuguilla* seeds from a contaminated site (Villa de la Paz, SLP) and from a noncontaminated site (Villa de Zaragoza, SLP) under eight cadmium concentrations. Different letters mean statistical differences between sites (*P* < 0.0001).

Treatment (mg/L Cd)	Seed germination (%)	
	Villa de Zaragoza	Villa de la Paz
0	87 ± 2.54^a^	31 ± 2.91^b^
1	76 ± 5.78^a^	27 ± 4.63^b^
10	73 ± 4.06^a^	25 ± 5.70^b^
20	77 ± 3.39^a^	26 ± 2.44^b^
40	72 ± 7.34^a^	29 ± 5.10^b^
60	72 ± 7.34^a^	29 ± 3.31^b^
80	71 ± 1.87^a^	22 ± 5.14^b^
100	73 ± 2.55^a^	24 ± 4.00^b^

**Table 3:** Radicle length ± standard error of *Agave lechuguilla* seeds from a contaminated site (Villa de la Paz, SLP) and from a noncontaminated site (Villa de Zaragoza, SLP) under eight cadmium concentrations. Different letters mean statistical differences between site X treatment factor (*P* < 0.0001).

Treatment (mg/L Cd)	Radicle length (cm)	
	Villa de Zaragoza	Villa de la Paz
0	2.06 ± 0.03^a^	1.64 ± 0.03^b^
1	1.79 ± 0.05^b^	1.07 ± 0.06^c^
10	1.49 ± 0.07^b^	0.91 ± 0.07^d^
20	1.4 ± 0.04^c^	1.09 ± 0.05^d^
40	1.3 ± 0.065^c^	0.97 ± 0.04^d^
60	1.4 ± 0.067^c^	1.11 ± 0.08^d^
80	1.24 ± 0.05^c^	0.93 ± 0.05^d^
100	0.98 ± 0.035^d^	0.98 ± 0.04^d^

## References

[1] Sauvé S, Hendershot W, Allen HE (2000). Solid-solution partitioning of metals in contaminated soils: dependence on pH, total metal burden, and organic matter. *Environmental Science and Technology*.

[2] Mohtadi A, Ghaderian SM, Schat H (2012). A comparison of lead accumulation and tolerance among heavy metal hyperaccumulating and non-hyperaccumulating metallophytes. *Plant and Soil*.

[3] Ye ZH, Baker AJM, Wong MH, Willis AJ (1997). Zinc, lead and cadmium tolerance, uptake and accumulation by *Typha latifolia*. *New Phytologist*.

[4] Cobb GP, Sands K, Waters M, Wixson BG, Dorward-King E (2000). Accumulation of heavy metals by vegetables grown in mine wastes. *Environmental Toxicology and Chemistry*.

[5] Lee CG, Chon H-T, Jung MC (2001). Heavy metal contamination in the vicinity of the Daduk Au-Ag-Pb-Zn mine in Korea. *Applied Geochemistry*.

[6] Vassilev A, Schwitzguebel J-P, Thewys T, van der Lelie D, Vangronsveld J (2004). The use of plants for remediation of metal-contaminated soils. *TheScientificWorldJOURNAL*.

[7] Cobbett C, Goldsbrough P (2002). Phytochelatins and metallothioneins: roles in heavy metal detoxification and homeostasis. *Annual Review of Plant Biology*.

[8] Akhter MF, McGarvey B, Macfie SM (2012). Reduced translocation of cadmium from roots is associated with increased production of phytochelatins and their precursors. *Journal of Plant Physiology*.

[9] Bianucci E, Sobrino-Plata J, Carpena-Ruiz RO (2012). Contribution of phytochelatins to cadmium tolerance in peanut plants. *Metallomics*.

[10] Gechev TS, Hille J (2012). Molecular basis of plant stress. *Cellular and Molecular Life Sciences*.

[11] Schat H, Llugany M, Vooijs R, Hartley-Whitaker J, Bleeker PM (2002). The role of phytochelatins in constitutive and adaptive heavy metal tolerances in hyperaccumulator and non-hyperaccumulator metallophytes. *Journal of Experimental Botany*.

[12] Küpper H, Mijovilovich A, Meyer-Klaucke W, Kroneck PMH (2004). Tissue- and age-dependent differences in the complexation of cadmium and zinc in the cadmium/zinc hyperaccumulator *Thlaspi caerulescens* (Ganges ecotype) revealed by x-ray absorption spectroscopy. *Plant Physiology*.

[13] Solti Á, Sárvári É, Tóth B, Basa B, Lévai L, Fodor F (2011). Cd affects the translocation of some metals either Fe-like or Ca-like way in poplar. *Plant Physiology and Biochemistry*.

[14] Gill SS, Khan NA, Tuteja N (2012). Cadmium at high dose perturbs growth, photosynthesis and nitrogen metabolism while at low dose it up regulates sulfur assimilation and antioxidant machinery in garden cress (*Lepidium sativum* L.). *Plant Science*.

[15] Razo I, Carrizales L, Castro J, Díaz-Barriga F, Monroy M (2004). Arsenic and heavy metal pollution of soil, water and sediments in a semi-arid climate mining area in Mexico. *Water, Air, and Soil Pollution*.

[16] Chapa-Vargas L, Monzalvo-Santos K (2012). Natural protected areas of San Luis Potosí, Mexico: ecological representativeness, risks, and conservation implications across scales. *International Journal of Geographical Information Science*.

[17] Romero-González J, Peralta-Videa JR, Rodríguez E, Delgado M, Gardea-Torresdey JL (2006). Potential of *Agave lechuguilla* biomass for Cr(III) removal from aqueous solutions: thermodynamic studies. *Bioresource Technology*.

[18] Sawalha MF, Peralta-Videa JR, Saupe GB, Dokken KM, Gardea-Torresdey JL (2007). Using FTIR to corroborate the identity of functional groups involved in the binding of Cd and Cr to saltbush (*Atriplex canescens*) biomass. *Chemosphere*.

[19] Demirezen D, Aksoy A (2006). Heavy metal levels in vegetables in Turkey are within safe limits for Cu, Zn, Ni and exceeded for Cd and Pb. *Journal of Food Quality*.

[20] Baskin C, Baskin JM (1998). *Seeds: Ecology Biogeography and Evolution of Dormancy and Germination*.

[21] Ruzin CE (1999). *Plant Microtechnique and Microscopy*.

[22] SAS Institute (1999). *SAS/STAT User’s Guide Version 8*.

[23] Sokal RR, Rohlf FJ (1994). *Biometry*.

[24] van der Ent A, Baker AJ, Reeves RD, Pollard AJ, Schat H (2013). Hyperaccumulators of metal and metalloid trace elements: facts and fiction. *Plant and Soil*.

[25] Kranner I, Colville L (2011). Metals and seeds: biochemical and molecular implications and their significance for seed germination. *Environmental and Experimental Botany*.

[26] Haque N, Peralta-Videa JR, Duarte-Gardea M, Gardea-Torresdey JL (2009). Differential effect of metals/metalloids on the growth and element uptake of mesquite plants obtained from plants grown at a copper mine tailing and commercial seeds. *Bioresource Technology*.

[27] Buendía-González L, Orozco-Villafuerte J, Cruz-Sosa F, Barrera-Díaz CE, Vernon-Carter EJ (2010). Prosopis laevigata a potential chromium (VI) and cadmium (II) hyperaccumulator desert plant. *Bioresource Technology*.

[28] Nieboer E, Richardson DHS (1980). The replacement of the nondescript term “heavy metals” by a biologically and chemically significant classification of metal ions. *Environmental Pollution B*.

[29] Clemens S (2006). Toxic metal accumulation, responses to exposure and mechanisms of tolerance in plants. *Biochimie*.

[30] Luković J, Merkulov J, Pajević S (2012). Quantitative assessment of effects of cadmium on the histological structure of poplar and willow leaves. *Water, Air and Soil Pollution*.

[31] Szalontai B, Horváth LI, Debreczeny M, Droppa M, Horváth G (1999). Molecular rearrangements of thylakoids after heavy metal poisoning, as seen by Fourier Transform Infrared (FTIR) and Electron Spin Resonance (ESR) spectroscopy. *Photosynthesis Research*.

[32] Ďurčeková K, Huttová J, Mistrík I, Ollé M, Tamás L (2007). Cadmium induces premature xylogenesis in barley roots. *Plant and Soil*.

[33] Donnelly JR, Shane JB, Schaberg PG (1990). Lead mobility within the xylem of red spruce seedlings: implications for the development of pollution histories. *Journal of Environmental Quality*.

[34] di Toppi LS, Gabbrielli R (1999). Response to cadmium in higher plants. *Environmental and Experimental Botany*.

[35] Drazic G, Mihailovic N (2005). Modification of cadmium toxicity in soybean seedlings by salicylic acid. *Plant Science*.

[36] Pomponi M, Censi V, di Girolamo VD (2006). Overexpression of *Arabidopsis* phytochelatin synthase in tobacco plants enhances Cd^2+^ tolerance and accumulation but not translocation to the shoot. *Planta*.

[37] Pernia B, Sousa A, Reyes R, Castillo M (2008). Biomarcadores de contaminación por cadmio en las plantas. *Interciencia*.

[38] Böhm PAF, Zanardo FML, Ferrarese MLL, Ferrarese-Filho O (2006). Peroxidase activity and lignification in soybean root growth-inhibition by juglone. *Biologia Plantarum*.

[39] Laszlo JA (1994). Changes in soybean fruit Ca^2+^ (Sr^2+^) and K^+^ (Rb^+^) transport ability during development. *Plant Physiology*.

[40] Stacey MG, Patel A, McClain WE (2008). The *Arabidopsis* AtOPT3 protein functions in metal homeostasis and movement of iron to developing seeds. *Plant Physiology*.

